# Using participatory epidemiology to investigate women’s knowledge on the seasonality and causes of acute malnutrition in Karamoja, Uganda

**DOI:** 10.1186/s13570-023-00269-5

**Published:** 2023-03-02

**Authors:** Andy Catley, Raphael Lotira Arasio, Charles Hopkins

**Affiliations:** Feinstein International Center, Friedman School of Nutrition Science and Policy, Tufts University, PO Box 6934, Kampala, Uganda

**Keywords:** Monthly calendar, Causal diagram, Agro-pastoralism, Indigenous knowledge, Pastoral women

## Abstract

The Karamoja region of northeast Uganda had been characterized by high levels of acute malnutrition (AM) for decades, despite substantial aid investment in nutrition programmes. Participatory epidemiology (PE) was used to understand the seasonality of child AM from the perspective of women agro-pastoralists and understand their knowledge and prioritization of the causes of child AM. Women provided highly plausible descriptions and analysis of monthly variations in the occurrence of AM, livelihood factors related to the temporal variation in AM occurrence, the root causes of AM and relationships between these causes. Overall, AM was strongly attributed to declining livestock ownership and access to cow milk and normalized gender discrimination. Monthly calendars revealed important monthly patterns in AM, births and women’s workload that had not been previously reported. There was significant agreement (*p* < 0.01) between independent women’s groups (*n* = 16) for the monthly calendars and causal diagrams, indicating strong reproducibility of the methods. Triangulation indicated good validity of the monthly calendar method. The PE approach demonstrated that agro-pastoralist women with limited formal education could describe and analyse the seasonality of AM and related factors and identify and prioritize the causes of AM. Indigenous knowledge should be valued and respected, and nutrition programmes should shift the emphasis towards far more participatory and community-based approaches. The timing of conventional nutrition surveys in agro-pastoral settings should be based on an understanding of the seasonality of livelihoods.

**Supplementary Information:**

The online version contains supplementary material available at 10.1186/s13570-023-00269-5.

## Introduction

### Malnutrition and changing livelihoods in Karamoja

The Karamoja Region in northeast Uganda is a remote semi-arid area inhabited mainly by agro-pastoralist and pastoralist communities. For decades, Karamoja has been characterized by economic and political marginalization, and relative to the rest of Uganda, it has low human development indicators (Catley et al. 2021). The human population is approximately one million people. In common with many other pastoralist areas of East Africa, Karamoja has high levels of acute malnutrition (AM). Acute malnutrition or wasting has a relatively rapid onset and recovery period relative to chronic malnutrition or stunting. It is most commonly identified in children aged 6 months to 5 years of age using the following anthropometric criteria: mid-upper arm circumference < 125 mm and/or weight-for-height *z* score of <  − 2 and/or bilateral pitting oedema. In 2016, 10% of children in Karamoja were wasted (low weight-for-height) compared to a national average for Uganda of 3.6% (Uganda Bureau of Statistics and ICF International, 2018). However, high levels of AM have persisted or worsened in Karamoja despite large-scale development programmes. In 2018, a review of global acute malnutrition (GAM) examined bi-annual GAM prevalence estimates in all districts of Karamoja between 2010 and 2017. During this period, GAM prevalence as measured by nutrition surveys in June each year increased from 11.5 to 13.8%; for nutrition surveys conducted in December each year, GAM increased from 9.8 to 10.4% (Government of Uganda et al. 2018). Simultaneously, there were numerous nutrition programmes in Karamoja during this period. For example, in 2016, there were 24 nutrition projects or programmes in Karamoja implemented by 17 organizations and with an emphasis on strengthening health systems, and social and behaviour change (Karamoja Resilience Support Unit 2016).

Major concerns about malnutrition and health in Karamoja are long-standing. In 1980, the region was affected by a famine that was attributed to multiple causes such as drought, livestock disease, cattle raiding, looting and disruption of markets and transportation (Biellik and Henderson, 1981). The reported overall human mortality was 21.2% but reached 60.7% in children under 1 year of age. From the mid-1990s, research on human adaptability in Karamoja included specific studies on child and maternal health, child growth and breastfeeding, and dietary change (Gray et al. 2008; Gray 2010; Gray and Sundal 2017). These studies explained child malnutrition by reference to a decline in agro-pastoralist production and falling livestock herd sizes, with associated reductions in animal milk availability and consumption. Declining herd size was attributed to livestock raids, livestock diseases and government-led disarmament activities. These studies also reported the multiple and increasing responsibilities of women as child carers, meal providers and income generators and the weak provision of health services. Very similar findings were reported in 2011 in a study that linked serious limitations in animal milk availability to insecurity, cattle raids and political marginalization (Stites and Mitchard 2011). Overall, the social science literature on Karamoja has consistently explained malnutrition by reference to pressures on agro-pastoralism and the importance of animal milk in the diet. In general terms, factors that cause a decline in livestock ownership and access to milk have direct impacts on the nutrition of mothers and children.

An important feature of Karamoja’s recent history was an intense and large-scale government disarmament programme between 2006 and 2011. This programme included the use of the Ugandan army to conduct armed air and ground attacks on civilians and forcefully restrain livestock in ‘protected kraals’. This practice reduced livestock access to pasture and water and was linked to atypical outbreaks of animal diseases and related mortality (Stites and Mitchard 2011). At the end of disarmament, Karamoja entered a period of relative peace and stability, livestock mobility increased and herds started to recover. By 2017, a very dynamic livestock trade had developed in the region, supplying animals to internal markets in Uganda, and cross-border markets in Kenya and South Sudan (Aklilu 2017). This evolving trade was widely seen as evidence of a growing economy in the region, yet as noted above, the prevalence of GAM increased between 2010 and 2017. One explanation for the co-existence of economic growth and rising malnutrition was that Karamoja was following a similar pattern of livelihood transitions to other pastoralist areas of east Africa over decades. In summary, combinations of human population growth, increasing commercialization of livestock systems, marked climatic variability and frequent droughts, weak governance and declining access to rangeland created a gradual redistribution of livestock from poorer to wealthier households (Catley and Aklilu 2013). While wealthier households were the main suppliers and beneficiaries of livestock markets, poorer households owned very few animals and relied heavily on diversified livelihood activities such as manual wage labour, which often required considerable effort for limited income (Catley et al. 2016). In Karamoja, there were indications that the disarmament programme had enabled a concentration of livestock ownership among wealthier owners (Stites et al. 2016), while poorer herders were pushed into non-livestock livelihood activities (Bushby and Stites 2016; Iyer and Mosebo 2017).

### Seasonality in pastoralist versus agrarian systems

It is widely recognized that agriculture systems often show marked seasonality, with distinct periods of land preparation, planting, weeding, harvesting and other activities, driven mainly by rainfall patterns. Food insecurity and malnutrition in agricultural areas of low-income countries often peak in the rainy period before the main harvest (Chamber et al. 1981). At this time of year, food stores from the previous year are running low, and food prices and levels of indebtedness are high. However, physical energy is needed for agricultural work. There can also be a high incidence of diseases at this time, such as malaria and diarrhoea, and women have increasing workloads.

Although this description of seasonality, food insecurity and malnutrition fits well with crop-based livelihoods, seasonality in many pastoralist areas of Africa is different. Rather than describing the main rainy season as a time of stress, pastoralists typically associate rain with times of plenty. This is when livestock produce the most milk, and milk consumption is associated with good nutrition and health (Sadler et al. 2009). In contrast to agrarian areas, pastoralists describe livelihoods and nutritional stress towards the end of long dry seasons. This is the time of year when livestock milk supply is declining, herders are forced to sell animals in exchange for cereals and livestock are moved long distances from home settlements to find grazing and water. Also, livestock prices tend to be low at this time because animals are in poor condition and cereals prices are high due to limited availability and high demand. This seasonality of pastoralist livelihoods partly explains why AM can peak in dry seasons and fall in wet seasons (Sadler et al. 2009).

Despite the fundamental importance of seasonality in pastoralist systems, up to 2018 the seasonal variation in malnutrition and contributing factors had not been described in Karamoja. The reasons for this were unclear but from the perspective of conventional food security and nutrition assessment (FSNA) surveys, there were probably operational and funding challenges to using these surveys each month during a 12-month period. Biannual FSNA surveys have been conducted in Karamoja in May/June and November/December since 2009 and provided point prevalence estimate of child malnutrition, as previously indicated. However, as these surveys did not cover other months the monthly and seasonal variation in GAM was unknown, with important implications for nutrition programming. For example, how could preventive measures for AM be delivered at the right time if the peaks in AM prevalence were not known?

### Participatory epidemiology

In the early 1990s, veterinarians in East Africa started to adapt participatory approaches and methods to investigate livestock diseases, especially in remote and conflict-affected pastoralist areas. An important aspect of the approach was professional recognition that pastoralists possessed strong indigenous knowledge on livestock production and diseases, including the clinical signs, modes of transmission and seasonality of diseases and vector populations. This indigenous knowledge had been reported by veterinarians since the 1950s (Mares 1954) and, over time, accounts from various pastoralists groups in different countries became available in the peer-reviewed and grey literature (Bizimana 1994). Veterinary uses of participatory methods became known as ‘participatory epidemiology’ (PE) and adaptations included the semi-standardization and repetition of PE methods that produced ranks, scores or proportions leading to datasets that could be analysed using conventional statistics (Catley et al. 2012). This approach led to estimates of disease incidence and mortality, analysis of seasonality and causation and an understanding of complex syndromes involving multiple infections (Catley et al. 1994). Veterinary researchers and epidemiologists continue to adapt and use PE, and the approach is supported by international agencies such as the World Organization for Animal Health and the Food and Agriculture Organization, particularly to support national livestock disease surveillance systems (Allepuz et al. 2017).

In human health and nutrition, there has been very wide and long-running use of participatory approaches and methods, across low-income to industrialized countries, and covering a vast range of contexts and aspects of health service provision, disease control and nutrition and dating back to the early 1990s (Rifkin et al. 1986). Similarly, community participation is viewed as an essential aspect of healthcare systems and most recently is reflected in the promotion and widespread use of community engagement frameworks for health service delivery (World Health Organization 2017). However, in pastoralist areas of East Africa, the use of participatory approaches and methods in the human health and nutrition sectors has been limited. Rare examples are available in the grey literature and include a participatory assessment of women’s health in southern Ethiopia (Tezera and Desta 2008) and studies on malnutrition in the Somali Region of Ethiopia (Sadler and Catley 2009).

Given the limited documentation of the seasonality of AM and related livelihood issues in Karamoja, we aimed to use PE to understand this seasonality from the perspective of women agro-pastoralists. We also aimed to understand women’s knowledge and prioritization of the causes of AM in children and mothers. From a methodological perspective, the work was exploratory because we aimed to assess if and how veterinary experiences with PE methods could be adapted for use in a study on human malnutrition.

## Methods

The study used two main stages. An initial ethnographic approach was used to understand the language used by agro-pastoralist women to describe malnutrition and related issues. Drawing on this local language and secondary literature, the second stage involved the semi-standardization of two PE methods, a monthly calendar and a causal diagram, and repetition of the methods with groups of women participants. The study was conducted in May and June 2018.

In the ethnographic stage, focus group discussions (FGDs) were conducted by a senior researcher (RLA) who was fluent in Ngakarimojong and who had extensive experience with informal interviewing techniques and in facilitating FGDs in Karamoja and other pastoralist areas of East Africa.

The PE methods were used by a team of facilitators comprising five women, all fluent in the local language and who received training in PE immediately before the fieldwork. These facilitators were supported by two male facilitators, both with more than 15 years of experience in using participatory methods in pastoralist areas of East Africa; one of these facilitators was also fluent in Ngakarimojong.

### Ethnographic stage

Focus group discussions (FGDs) were used to document the language used by women to describe malnutrition and related issues, such as the causes of malnutrition. The FGDs were structured around a checklist of issues and covered the words and terms in Ngakarimojong for: seasons and months; different types of malnutrition in children and adults, including AM and different types of health, illness and well-being.

The ethnography was conducted in Amudat, Moroto, Kaabong and Kotido districts with 16 groups of women participants and with group size ranging from five to eight women. The districts were selected based on the predominance of agro-pastoralism. Within each district, two sub-counties were purposively sampled and within each sub-county, two villages were purposively sampled.

### Participatory epidemiology stage: monthly calendar

A monthly calendar is a visualization method showing the pattern of selected ‘indicators’ by month. As literacy rates among women were low in Karamoja, the method used pictures or local objects to depict the months and indicators, and all guidance from the facilitators on the method and all discussion was conducted verbally. This approach aimed to ensure the involvement of all women in a group irrespective of their formal literacy level. A detailed account of the monthly calendar method is provided as Additional file 1 (field guide for monthly calendar method), and key aspects of the method are described below.

The calendar was constructed on the ground, using diagrams to represent months and other diagrams to represent the indicators (see Additional file 1, Fig. 1). Ngakarimojong names for months are shown in the Additional file 1: Table S8. Each time a diagram was used, its meaning was explained to the participants, and their understanding of the diagram was checked. The diagrams depicting months are arranged on the ground in a row, and again, the meaning of each diagram was explained and checked.

Rainfall was selected as the first indicator and the participants were asked to show the pattern of rainfall against each month. This was done by asking them to distribute a pile of 100 stones against the months and with guidance that all of the stones had to be used. Then, participants were also asked to consider a ‘typical year’ rather than a year with abnormally low rainfall from their perspective. The distribution of the stones was a group process. Participants discussed the task and collectively decided on how to arrange the stones. After the rainfall pattern was ‘scored’, participants were asked questions to check their understanding of the method and ensure that the pattern of monthly rainfall shown was as they intended.

After rainfall had been scored, the stones were left in place, and further indicators were scored, one by one, with discussion and further questions for each indicator. Ten further indicators were selected based on the ethnographic work and literature review. This number of indicators was also considered to be appropriate in terms of the amount of time we felt that women should spend on the method. The full list of indicators was as follows: rainfall; availability of cow milk for children; availability of goat milk for children; availability of own-produced sorghum; women’s work time in their own gardens; women’s other work time, especially work for income; occurrence of AM in children; occurrence of child malaria; occurrence of child diarrhoea; and occurrence of human births. To make the child malnutrition indicator age-specific, women were asked to focus on a 2-year-old child. This age of the child was used because nutrition programme strategies in Karamoja were often framed around the first 1000 days after a child was born, in line with global strategies of organizations such as the United Nations Children’s Fund (UNICEF 2020). After the scoring of each indicator by month, questions were asked to probe the scores and understand the women’s reasoning behind their scoring. A completed monthly calendar showed the patterns of all 10 indicators and was used as the basis for further questions, particularly questions on the relationships between indicators.

In addition to the use of questions to probe the monthly calendar results, a PE method called proportional piling was used as a rapid method for quantifying responses related to the availability of livestock milk. Participants were asked to distribute a pile of 100 stones to show the pattern of households with sufficient access to cow milk versus those with insufficient access. The method was repeated for goat milk.

The monthly calendar was repeated with 16 independent groups of women, 8 in Moroto District and 8 in Kotido District; these groups were different to those who were involved in the ethnographic stage. In each district sub-counties and villages were selected purposively with preference given to more rural sub-counties. From a total of 5 sub-counties in Moroto District, 4 sub-counties were selected viz. Nadunget, (3 villages), Katikekile (1 village), Rupa (2 villages) and Tapac (1 village); the Moroto Town Council administrative area was also selected (1 village). From a total of 6 sub-counties in Kotido District, another 4 sub-counties were selected viz. Nakapelimoru (1 village), Panyangara (3 villages), Kacheri (1 village) and Rengen (1 village); the Kotido municipality was also selected (2 villages). In each selected village, the Local Council chairman (village-level head person) was visited to explain the purpose of the study and the need to select a group of women to participate. The criteria for selection were discussed, and the chairman then selected a local woman as a participant but also to work with the team’s female research assistants to identify the other participants. Each group included lactating and pregnant women, at least one elderly woman, and the traditional birth attendant. Each group was in a different sub-county and a different village, using a purposively selection of sites. Group sizes varied from eight to 12 women.

Although the monthly calendar was a visualization method, the use of 100 counters per indicator enabled the results to be recorded numerically. This numerical record was complemented with written notes from the follow-up questions. The scores for each month and indicator from the 16 participant groups were summarized using the median score in MS Excel.

To assess the reproducibility of the monthly calendar method across the 16 participant groups, Kendall’s coefficient of concordance (*W*) was used in the SPSS software (SPSS Inc. 2011). According to the description of this statistic by Siegal and Castellan (1988), the level of agreement between the groups was categorized as ‘weak’, ‘moderate’ or ‘strong’ based on the *p*-values assigned to *W* values by the SPSS software.

To assess the validity of the monthly calendar method, two main approaches were used, depending on the indicator in question. For rainfall, the results from the monthly calendar method were compared with objective measures of rainfall, using monthly rain gauge data from Namalu in Karamoja between 1947 and 1987; this was the most comprehensive and long-term set of rainfall data that was available for the region. For other indicators, the technical plausibility of the results was assessed by reference to the literature on the indicator in question.

### Participatory epidemiology stage: Causal diagram

The causal diagram method was used to further understand the main causes of AM in children as described by participants, the relative importance of these causes and any relationships between different causes. During the ethnographic stage of the study, women had mentioned specific causes of AM, and these causes were used in the causal diagrams. The same causes were further described by women, at length, during the monthly calendar method. For the causal diagram, simple diagrams were used to represent each cause and a malnourished 2-year-old child. The meaning of each diagram was explained by the facilitators, using Ngakarimojong terms. The diagrams of causes were then placed on the ground in a circle around the diagram of the child in the centre and women were asked to show the relative importance of each cause using a pile of 100 stones. The visualization and scoring of the causes then enabled further questions and discussion on the relative importance of the causes and how different causes related to each other sequentially or otherwise, enabling some causes to be clustered.

The causal diagram method was repeated with the same 16 independent participant groups as for the monthly calendar method. The scores for each cause were summarized using the median score, and reproducibility was assessed using *W.*

## Results

### The language of malnutrition

The ethnographic stage of the study produced detailed Ngakarimojong words and terms related to AM in children and mothers, including language for children and adults by age and gender; child size and child growth; children with different health conditions; different causes of malnutrition; mothers at different stages of pregnancy, birth and lactation; and mothers with different health conditions and with malnutrition linked to different causes. Detailed lists of Ngakarimojong words are available as Additional file 1, but there were two important general findings. First, women had a rich and detailed vocabulary related to malnutrition and disease, as well as specific age groups of children, and stages of pregnancy, birth and lactation. Women described different types of child malnutrition and used specific terms for ‘malnourished due to poor diet’ and ‘malnourished due to food insufficiency’, and so they distinguished between diet quality and diet quantity. They distinguished between wasting (low weight for height) and stunting (low height for age) and also used specific terms for malnutrition due to disease, non-spaced pregnancies, poor quality breast milk, inadequate breast milk and due to a poor relationship between parents. Second, although Ngakarimojong was used in all three districts, there were numerous dialectic differences or preferences related to the language used. The facilitators took these differences into account as they conducted the research in different areas.

### The seasonality of malnutrition and related factors

**Fig. 1:**
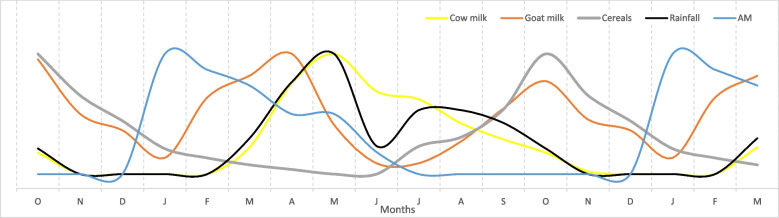
Monthly pattern of food availability, rainfall and acute malnutrition in children. *Note*: The graph is derived from monthly calendars with 16 independent women’s groups and median scores; the monthly calendar method used a 12-month period; the graph shows an 18-month period to clarify the trends between December and January. AM, acute child malnutrition

**Fig. 2:**
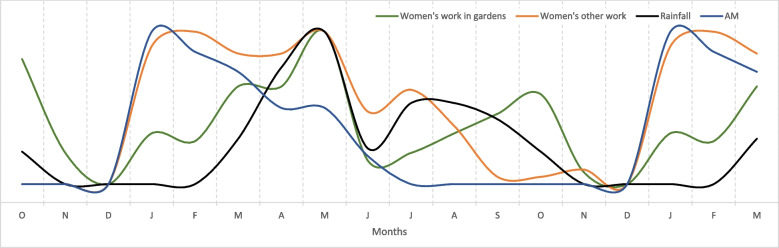
Monthly patterns of women’s work, rainfall and acute malnutrition in children. *Note*: The graph is derived from monthly calendars with 16 independent women’s groups and median scores; the monthly calendar method used a 12-month period; the graph shows an 18-month period to clarify trends between December and January. AM, acute child malnutrition

**Fig. 3:**
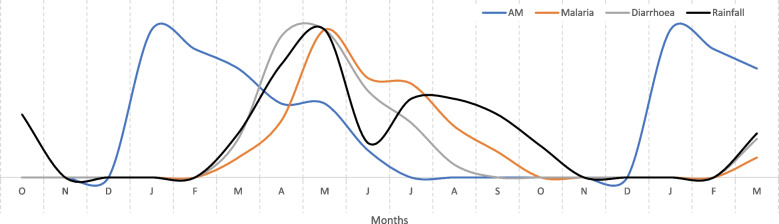
Monthly pattern of malaria, diarrhoea, rainfall and acute malnutrition in children. *Note*: The graph is derived from monthly calendars with 16 independent women’s groups and median scores; the monthly calendar method used a 12-month period; the graph shows an 18-month period to clarify trends between December and January. AM, acute child malnutrition

**Fig. 4:**
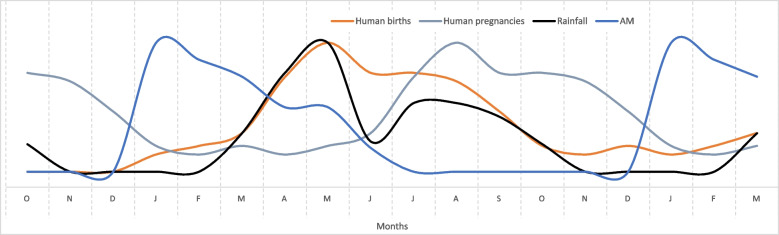
Monthly patterns of pregnancies, births, rainfall and acute malnutrition in children. *Note*: The graph is derived from monthly calendars with 16 independent women’s groups and median scores; the monthly calendar method used a 12-month period; the graph shows an 18-month period to clarify trends between December and January. The monthly calendar did not include an indicator for pregnancy and so the pregnancy pattern was derived from the birth indicator. AM, acute child malnutrition

In PE, there is no standard way to present the results from monthly calendars, and in this study, the median scores from the 16 monthly calendars were used to produce line graphs. To prevent too much information (too many lines) being shown on a single graph, four graphs were used but with each graph showing rainfall and AM as common indicators (Figs. 1, 2, 3, and 4). The graphs lack a *y*-axis scale because although 100 stones were used to score each indicator against each month, this number was arbitrary and had no absolute meaning. When a line shows a peak, the peak represents the highest level of that indicator during the year; when a line shows a trough this represents the lowest level of the indicator during the year, although this level might not necessarily be zero in absolute terms. Each graph also shows an 18-month period on the *x*-axis to clearly show monthly patterns at the beginning and end of the year.

Figure 1 showed a bi-modal rainfall pattern in a typical year with a peak in rain in April/May and a second but lower peak in July/August; there was a long, 4-month dry season from November to February. Observations of AM in children increased sharply in December and peaked in January, about halfway through the dry season. With the onset of rain in late February, observations of AM decreased and continued to fall, reaching the lowest levels between July and November. As outlined above, these lower levels of AM were not interpreted as zero AM but as lower levels relative to the peak in January.

The peak in AM coincided with the lowest availability of staples foods, viz. own-produced animal milk and sorghum (Fig. 1). The availability of cow milk closely followed the rainfall pattern because the grazing system for cattle relied on rainfed pastures, and seasonal movements to access these pastures. Low availability of cow milk in the dry season was attributed to cows being in the kraals (mobile camps), insufficient water for cattle, poor quality of grass and animal reproduction factors such as pregnancies and calf growth. In general, migration to kraals began in October/November, but in areas with less grazing, migrations began as early as August or September. The only families in the villages that had milk in the dry season were those that kept a few cows nearby and used supplementary feeding with tree products or crop residues or those that had preserved milk (e.g. ghee). Cows that were judged to be hardy were purposively selected to remain in the villages, but not everyone had access to the milk from these animals. Families with no access to milk in the dry season fed their children with poor diets such as tea and porridge without milk.

Overall, the limited access to cow milk for many households was associated with low cattle ownership; a median proportion of only 18% of households was categorized as having sufficient access to cow milk. In addition, dry season access to milk was restricted for women and children because they remained in homesteads when cattle were moved to distant grazing areas. In part, this was because women were responsible for important home-based tasks during the dry season (see later, and also the Additional file 1). The availability of goat milk reflected different management practices for goats relative to cattle. By using staggered breeding practices goats were managed to try to produce milk from January to April, being the period when cattle were away in dry season grazing areas or migrating back to homesteads. Other goats were managed to produce milk between September and December, when cow milk production was declining. However, in common with cattle, the overall ownership of goats was low and only a median proportion of 23% of households was categorized as having sufficient access to goat milk. During the year, sorghum first became available in July/August, with increasing harvests in October with a decline through to December. However, the absolute amount of sorghum produced was low, and this was attributed to small plots, called ‘gardens’ locally, and production systems that relied heavily on manual labour.

The discussion on cattle ownership and milk access included women’s accounts of how declining cattle ownership was changing household practices, leading to increasing pressures on women. Notably, women were seen as responsible for sourcing food and preparing family meals, and a decline in cow milk access forced them to rely more on other sources of own-produced food, especially cereals, and seek additional income to buy food. Both practices placed an additional work burden on women and negatively affected childcare. At the same time, a failure to provide sufficient food placed women at risk of domestic abuse, including violent beatings by their husbands. This type of narrative was repeated by participants across groups and showed that while both seasonal patterns and an absolute limitation in cow milk availability were concerns, these factors were drivers of wider changes.

In Fig. 2, women’s work in their gardens started with land preparation with hand hoes before the onset of rain, followed by planting, weeding, harvesting, threshing and winnowing. A more detailed account of this work by month is provided in Additional file 1. Women’s other work covered all other tasks including childcare, cleaning the home and food preparation, as well as income generation activities such as harvesting and processing *Aloe vera*, working in mines, causal labour in towns, the collection and sale of firewood, charcoal production and sales, and producing and selling local beer. As the availability of own-produced foods was limited during the mid to late dry season (Fig. 1) women were compelled to find income for food purchases at this time, and the period of maximum ‘other work’ was January to May, with a decline as own-produced foods became available. The period of particularly heavy workload coincided with the peak in AM. Participants explained that work in gardens and to earn income often required long period away from home, during which young children were looked after by siblings or grandparents, if available.

Figure 3 shows that observed cases of malaria and diarrhoea coincided with the pattern of monthly rainfall, with relatively few cases observed during the dry season and when AM peaked in incidence. This finding indicated that although malaria and diarrhoea might be causes of AM in the wet seasons, or exacerbate AM due to other causes at these times of year, this was far less likely for the dry season peak in AM.

Figure 4 shows monthly patterns of human pregnancy, childbirth, AM and rainfall. Pregnancy occurred mainly between June and December, leading to births from March to September. The main birth period correlates closely with rainfall and the availability of cow milk (Fig. 1). Some early pregnancies occurred in April/May as men return from kraals at the end of the dry season; some milk is also available. In July/August, most courting takes place, women’s work in their own gardens tends to decline, and there are milk available and traditional dances. In October/November, there is high cereal availability and many ceremonies such as marriages take place.

The correlation between births and rainfall indicates that most births occur in the rainy season when cow milk and grain are available. In theory, this is good for the breastfeeding mother and the newborn child from the perspective of the mother’s diet and the quality and supply of breast milk. In practice, low livestock ownership leads to insufficient access to milk throughout the main birth months, even though milk supply peaks at this time of year. In addition, women’s workload is high during this period (Fig. 2).

Table 1 shows the level of agreement between the 16 participant groups for their scores for each indicator in the monthly calendar. There was strong agreement between the groups for 9/10 indicators. Weak agreement for the availability of goat milk was explained by different goat management practices in different areas and between different households, leading to variations in the monthly availability of goat milk.

**Table 1:** Level of agreement between independent participant groups for monthly calendars (*n* = 16)

Monthly calendar indicator	Kendall’s coefficient of concordance *W*	Level of agreement
Rainfall	0.73 (*p* < 0.001)	Strong
Availability of cow milk	0.86 (*p* < 0.001)	Strong
Availability of goat milk	0.10 (*p* > 0.05)	Weak
Availability of own-produced sorghum	0.80 (*p* < 0.001)	Strong
Women’s work in their own gardens	0.59 (*p* < 0.001)	Strong
Women’s other work	0.46 (*p* < 0.001)	Strong
Child malnutrition	0.60 (*p* < 0.001)	Strong
Child malaria	0.83 (*p* < 0.001)	Strong
Child diarrhoea	0.72 (*p* < 0.001)	Strong
Human births	0.50 (*p* < 0.001)	Strong

**Fig. 5:**
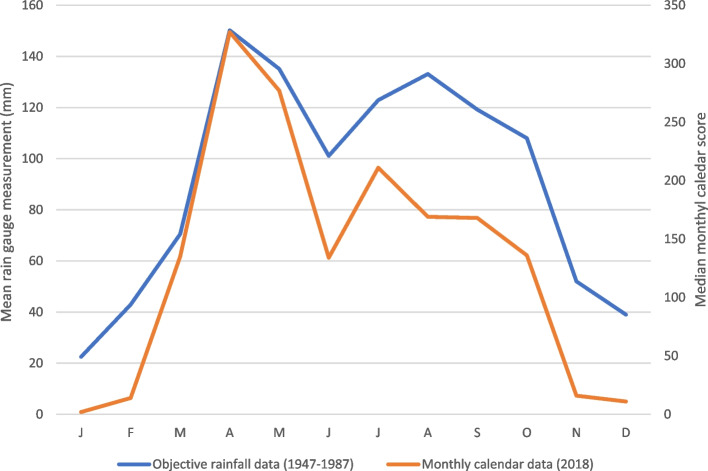
Objective rainfall data and monthly calendar rainfall pattern, Karamoja

Table 2 shows how secondary data and literature were used to triangulate the results for each indicator in the monthly calendars and provides statements on the technical plausibility of the monthly patterns. For monthly rainfall patterns, monthly calendar results are compared with objective measures of rainfall in Fig. 5.

**Table 2:** Triangulation of monthly calendar results and assessment of technical plausibility

Indicator	Triangulation
Rainfall	Comparison with objective rainfall data (Fig. 6) and literature (Chaplin et al. 2017) shows strong agreement Monthly calendar results were highly plausible
Availability of cow milk	Animal production literature explains how cow milk production in African pastoralist systems follows rainfallpatterns and availability of pasture (Jahnke 1982). Monthly calendar results were highly plausible
Availability of goat milk	Animal production literature explains how goat milk production in African pastoralist systems follows rainfall patterns, breeding management and availability of browse (Devandra and McLeroy 1982). Monthly calendar results were highly plausible
Availability of own-produced sorghum	Agriculture literature explains the seasonality of the rain-fed crop production, including sorghum production in dryland areas of Africa and specifically in Karamoja (Robinson and Zappacosta 2014). Constraints to sorghum production in Karamoja explain low production (Cullis 2018). Monthly calendar results were highly plausible
Women’s work in own gardens	Monthly calendar results highly plausible if compared to the pattern of sorghum production (see above) and related tasks; women carry out most of the work in gardens in Karamoja (Cullis 2018)
Women’s other work	Monthly calendar results highly plausible if monthly patterns of staple, own-produced foods are valid (see above) Women’s labour in Karamoja has been described in detail (Iyer and Mosebo 2017)
Child acute malnutrition	Limited monthly data available for triangulation. Monthly patterns in child AM highly plausible if patterns of food availability, women’s workload and birth patterns are used to explain AM
Child malaria	Limited monthly health data available for triangulation Vector populations expected to increase during wet seasons with related peaks in disease incidence (Selvaraj et al. 2018) Monthly calendar results highly plausible
Child diarrhoea	Limited monthly health data available for triangulation Diarrhoea caused by bacteria and *Cryptosporidium* had a higher prevalence in rainy/summer months in the tropics compared to dry/summer months; rotavirus prevalence was higher in drier months (Chao et al. 2019). Households had limited access to clean water or sanitation in study areas Monthly calendar results are plausible if the main infectious causes of diarrhoea in the study areas are bacteria and *Cryptosporidium*
Human births	Limited monthly health data available for triangulation Marked seasonality of births reported in neighbouring pastoralist area of Kenya, with the same pattern as study sites (Leslie and Fry 1989)

### Causes of malnutrition

**Fig. 6:**
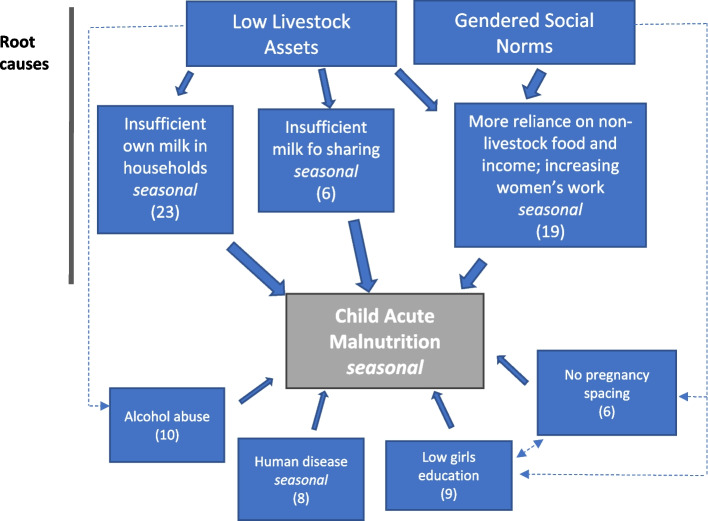
Participatory causal diagram for child acute malnutrition, Karamoja. *Note*: The figure derived from causal diagrams produced by 16 independent groups of women participants. Figures in parentheses are median scores. Causes that showed marked seasonal variation are annotated as ‘seasonal’. There was strong agreement between the participant groups on their scoring of the causes; *W* = 0.62 (*p* < 0.001)

A summarized causal diagram (Fig. 6) showed similar results to the monthly calendars in terms of the prominence of limited livestock ownership and gender norms as root causes of AM. As with the monthly calendars, these two causes were linked because as livestock holdings declined, women faced increasing domestic pressure to find non-livestock sources of food and income. Traditionally men played major roles in livestock ownership and management but falling livestock ownership was associated with negative behavioural changes such as increasing alcohol consumption. This increased the risk of gender-based violence, especially if limited food was prepared in the household; participants described increasing levels of psychological stress. Issues related to women’s workload were discussed under gendered social norms and similar issues were raised by participants during the monthly calendar method. Also related to gender, women described the challenges of pregnancy spacing and limited education of girls, and the linkages between education and pregnancy. The issue of pregnancy spacing was explained by reference to women having limited power to resist their partner’s demands and the risk of violent beatings. Disease was also noted as a cause of AM.

Participants also described variations in women’s knowledge and behaviours related to AM and, for example, gave accounts of mothers they viewed as irresponsible due to excessive alcohol use or due to a failure to plan properly for childcare. However, these behaviours were discussed within the wider context of food availability, the considerable level of effort needed to acquire income but with low levels of remuneration, and the fear of domestic abuse. These discussions led to detailed accounts from participants on the diets of ‘healthy’ versus ‘malnourished’ women during pregnancy and lactation as compiled in Tables 3 and 4. Notably, participants not only provided details of food types but also social, wealth and behavioural characteristics.

**Table 3:** Diets and other factors associated with healthy vs. malnourished pregnant mothers

	Healthy pregnant mother	Malnourished pregnant mother
Diet	Milk—fresh and sour Beans and butterbeans Eggs Chicken Local brew (*kwete*) Bananas Wild greens Salt Butter Sorghum porridge Meat (*akiring*) Milky tea Irish potatoes Onions Tomatoes Rice	Plain porridge (*ngakima*); no sugar added) from millet or sorghum Wild greens (plain), only salt added Wild tubers (plain) Wild fruits (plain) Sorghum flour (plain) Local brew (*ngagwe*)
Other factors	No disease	Poor family: no animals; no money to buy good-quality food Eating just once or twice a day Diseases such as malaria

**Table 4:** Diets of healthy vs. malnourished lactating mothers

	Healthy lactating mother	Malnourished lactating mother
Diet	Plain milk Sour milk Milky tea (*echai*) Fresh meat and meat soup Preserved meat Porridge (*uji*) with milk and butter Chicken Tomatoes and butter Local brew (*ngagwe/ebutiya*) Beans Wild vegetables such as *ekorete*, *eboo*, *ekiliton*, *ebolochete* and other ingredients, e.g. butter Sorghum flour with vegetables and melon Eggs	Local brew (*kwete*) Plain sorghum porridge Wild vegetables Watermelon Beans (plain), sometimes only when provided by friends and relatives Chicken—only if provided by friends and relatives Occasionally other foods if there is money to buy them General food insufficiency
Other factors	Responsible husband	Disease

## Discussion

### Monthly variations in acute malnutrition and related factors

The monthly calendar method used in this study was adapted from seasonal calendars that were widely used in social science research, rapid rural appraisal and participatory rural appraisal from the late 1980s to show the seasonality of livelihood activities, food sources and other information. Dryland examples include a seasonal calendar developed more than 30 years ago in South Darfur, Sudan, showing the seasonal availability of foods (Kerven 1987), and similarly, a seasonal calendar from North Kordofan in Sudan showed seasonal patterns of rainfall, crop production, labour demands and labour migration, use of different water sources and sorghum prices (Theis and Grady 1991). Seasonal calendars have also featured regularly in livelihood profile reports from pastoralist areas of East African countries, including Ethiopia (Disaster Prevention and Preparedness Agency and Save the Children 2002), Kenya (Famine Early Warning Systems Network 2010), Somalia (Food Security and Nutrition Analysis Unit 2016) and South Sudan (Muchombo and Sharp 2002).

In the adapted method used in this study, we aimed to improve the seasonal calendar in five ways: we used months not seasons to support more temporal specificity; the scoring of indicators enabled the relative size of each indicator to be measured (rather than simply assessing whether an indicator was present or not in a given month); the method was semi-standardized and repeated with independent participant groups; the level of agreement between participant groups was measured using a statistical test (*W*); and the results were triangulated against secondary data or literature (Table 2).

Looking in detail at triangulation, Fig. 5 does not show an exact match between objective rainfall measures and the monthly calendar rainfall pattern. However, the objective rainfall data was collected from a single location in the far south of Karamoja, outside the study areas, and over a different period to the monthly calendar data. For AM, malaria, diarrhoea and birth indicators we attempted to triangulate the monthly patterns using records from health clinics. However, these records depended heavily on user attendance at the clinics and attendance was likely to be affected by the availability and accessibility of services. In cases where clinics had insufficient medicines or staff (low service availability), usage would be low. This situation would arise if supplies of medicines or materials to the clinics were irregular and determined by budgets and bureaucracy rather than demand. Similarly, accessibility would be affected by the seasonal movement of livestock herds and people, with relatively low accessibility to fixed-point clinics during the dry season. For these reasons, we judged clinic records to be a measure of clinic performance rather than providing valid estimates of disease or birth incidence. Overall, based on the assessment of reproducibility (Table 1) and technical plausibility (Table 2), the monthly calendar method was judged to be reliable and valid.

The two main limitations of the monthly calendar method were that it did not produce absolute measures of each indicator by month, and the number of indicators was limited to 11; it was assumed that more indicators would make the method too time-consuming. Also, through the use of follow-up questions, each calendar produced a substantial amount of narrative data from each participant group, covering their reasons and explanations for the monthly patterns. When summarizing this data in an academic paper, there is a risk that the need for brevity prevents a full account of the participant’s perspectives. The methods used in the study focused on a 2-year-old child, and therefore, the results do not necessarily apply to other age groups.

The monthly calendars produced information on three types of monthly variation which to our knowledge had not been previously reported for Karamoja. First, the results clearly showed the pattern of AM by month and a peak in AM in the dry season (Fig. 1) that was not captured in bi-annual FSNA surveys. For these surveys to detect maximum and minimal levels of AM, the timing of the surveys should be revised to take place in January and September each year. In general, the monthly calendar method could be a useful complement to conventional quantitative surveys in Karamoja and more widely could also strengthen approaches to research and nutrition causal analysis that use limited community participation. For example, the PE approach showed that reliable and valid information on seasonality could be gathered in a participatory way, and far quicker, less expensive and more ethically than quantitative longitudinal studies.

Second, the monthly calendars clearly showed how peaks in women’s work coincided with peaks in AM (Fig. 2). In the mid-dry season when livestock milk was in very short supply and when the previous year’s store of sorghum was running low, women were obliged to find food and commonly resorted to income-generating activities that required considerable physical effort and time, but for very limited income. These activities have been described in detail in Karamoja, as has the importance of women’s paid labour during the dry season (Iyer and Mosebo 2017). However, daily wage rates were as low as USD 0.28/day for domestic work and USD 0.56/day for causal labour.

Third, there was marked seasonality of births in the study sites (Fig. 4), and this result fits with a far wider analysis showing that seasonality of birth in sub-Saharan Africa (and other regions) is common (Dorelian 2013). In the Karamoja, case births were very closely aligned with rainfall patterns and the availability of cow milk (Figs. 1 and 4). This birthing pattern could be a successful human adaptation when cattle ownership and milk supply are sufficient. However, reports of declining cattle ownership and pressures on agro-pastoralism in Karamoja date back to 2008 (Gray et al. 2008) and since then, research has continued to describe the impacts of these changes on diets (Stites and Mitchard 2011; Gray and Sundal 2017). Recent research on livestock ownership in Karamoja categorized 56.5% of households as livestock-poor, with insufficient animals to function as agro-pastoral units (Catley and Ayele 2021). This was consistent with declines in per capita ownership of livestock in many other pastoralist areas of East Africa, which were attributed to combinations of human population growth, reduced access to grazing land, commercialization of livestock production, frequent droughts and conflict, and other factors (Catley et al. 2016). Birth patterns in Karamoja indicate that most children were born when cow milk availability peaked from a seasonal perspective (Figs. 1 and 4), but with an overall deficit in milk availability due to limited cattle ownership. Notably, the diets of malnourished lactating women were described as lacking milk or meat, unless chicken was provided by relatives or friends (Table 4). The peak in births also coincided with peaks in malaria and diarrhoea (Figs. 3 and 4), indicating that many women in late pregnancy or soon after childbirth were exposed to these diseases.

### Weighting the perceived causes of acute malnutrition

The causal diagram shows that women explained AM mainly in terms of the limited availability of livestock and milk, and social norms that make them overburdened with the work of childcare and finding food for the family (Fig. 6). These two main causes of malnutrition were interlinked and cascaded down into various other issues and problems. In particular, the limited livestock ownership has a direct impact on food availability because milk supply was low, and this forced women to find more non-livestock sources of food and income. These non-livestock activities included crop production, but as described by Cullis (2018), plots were small and there was a high risk of rain failure. As noted above, various other non-livestock activities often involved considerable effort for limited reward and hindered childcare. This situation created another layer of nutritional risk for unweaned children, because women associated excessive work with poor-quality breast milk, which they linked to poor child nutrition. Further problems had roots in livestock-gender issues such as the loss of cattle affecting men’s self-identity and sense of purpose, men spending more time in villages than in the past and more consumption of local brew and hard liquor. Other research in Karamoja has described the substantial inflow of inexpensive alcohol into the region and the negative health and social consequences (Iyer et al. 2018). In turn, this led to even more violence towards wives and short intervals between pregnancies. For women, increasing workloads, the stress associated with finding food and income, and the risk of violence from husbands also lead to alcohol abuse.

The use of adapted PE methods showed how the seasonality of AM in Karamoja peaked in the dry season as the availability of own-produced staple foods declined. From a livelihoods perspective, women requested assistance to reduce AM such as the provision of livestock (restocking) and income generation opportunities that enabled childcare. Their preferences contrasted with preventive nutrition programmes in the region which focused on strengthening health centre capacities and social and behaviour change activities; the latter assumed that women’s knowledge on nutrition was limited. The PE approach showed how gender issues were prominent causes of AM and were prioritized and clearly explained by women. In terms of aid programmes, women described their involvement in gender-related behaviour activities but noted that men were not often targeted.

This work was conducted in 2018 and was partly a response to reports of worsening levels of AM in Karamoja despite high aid investment in nutrition programmes. This was also a period of relative peace and stability in the region, which followed a government disarmament programme that ended in 2011. However, in March 2020, the Ugandan government established COVID-19 lockdown measures nationally, which included market closures and travel restrictions. These measures had major impacts on livelihoods and food security in Karamoja, as livestock could not be sold, opportunities for earning income declined, and supply chains were disrupted (Arasio et al. 2020). Food prices increased dramatically, as did the cost of inputs needed to support livestock and crop production. The combination of increasing hunger and the limited presence of police or security forces also led to a resurgence in armed cattle raiding. These trends indicate that levels of AM in Karamoja have likely risen since 2018, and if so, there is an urgent need to revisit the causal logic of malnutrition prevention programmes in the region and design programmes with far greater community participation.

## Conclusions

The use of adapted PE methods was a reliable and valid approach for understanding the seasonality and causes of AM in a remote and under-developed agro-pastoral setting. The study revealed monthly patterns of AM, women’s workload and births that to our knowledge had not been previously reported. PE could complement conventional methods and approaches used by nutritionists such as quantitative surveys and nutrition causal analysis and could play a useful role in the design of programmes to prevent malnutrition.

## Supplementary Information

**Additional file 1.** Supplementary figure and tables.

## Data Availability

The datasets used and/or analysed during the current study are available from the corresponding author upon reasonable request.
