# Supplementary material for: Using participatory epidemiology to investigate women’s knowledge on the seasonality and causes of acute malnutrition in Karamoja, Uganda
Source: Pastoralism. 2023 Mar 2;13(1):7. doi: 10.1186/s13570-023-00269-5 (PMC9977474; doi:10.1186/s13570-023-00269-5)
Supplement: Supplementary file 1 — Additional file 1. Supplementary figure and tables. [file 13570_2023_269_MOESM1_ESM.docx]

**Additional files**

Contents

[Field guide for monthly calendar method 2](#_Toc106359278)

[Figure 1. Example of a monthly calendar being prepared. 5](#_Toc106359279)

[Table S1. Ngakarimojong language for children and adults by age and gender 6](#_Toc106359280)

[Table S2. Ngakarimojong language for child size and growth 7](#_Toc106359281)

[Table S3. Ngakarimojong language for children with different health conditions and different causes of malnutrition 8](#_Toc106359282)

[Table S4. Ngakarimojong language for mothers at different stages of pregnancy, at birth, at different stages of lactation 9](#_Toc106359283)

[Table S5. Ngakarimojong language for pregnant mothers with different health conditions and different causes of malnutrition 10](#_Toc106359284)

[Table S6. Women’s activities and times in own gardens by month 11](#_Toc106359285)

[Table S7. Illustrative women’s work activities and time spent 12](#_Toc106359286)

##

# Field guide for monthly calendar method

1. Preparation

Before going to the field,

- 1. Collect information on local terminology for months, and be ready to use this terminology.
  2. Prepare diagrams to represent each month, and be ready to explain these diagrams to the informants.
  3. Prepare diagrams to represent the indicators for the seasonal calendars, or use objects to represent the indicators. Be ready to explain the meaning of each diagram or indicator.
  4. Have several bags of counters ready, with bag containing 100 counters (e.g. 100 stones).

Key point – most or all informants will be illiterate. Use diagrams and verbal dialogue; do not use any written words.

1. Make a 1-year line by month

The method is done on the ground. Make a long line on the ground to represent a “typical year”. Place the diagrams for each month along the line, and:

- 1. Explain the meaning of the line and the diagrams to the informants
  2. Check their understanding e.g. point to a diagram and ask *“Which month is this?”*
  3. If there is any misunderstandings, explain the diagrams again, and until there is a good understanding of the diagrams/months.

1. Scoring of Rainfall – the first indicator
   1. Select “Rainfall” as the first indicator.
   2. Place the Rainfall diagram on the left side of the months, and explain the meaning of the diagram.
   3. Select a bag of 100 counters. Ask the group to distribute the counters to show the pattern of rainfall by month. A month with a lot of rain should be given a lot of counters, whereas a month with no rain would have no counters. The group should use all 100 counters.
   4. Leave the group for about 5-10 minutes to distribute the counters. Only intervene if the group need clarification of the method.
   5. During the scoring there will be a lot of discussion among the group. Listen carefully to the discussion, but do not interfere. Key issues should be recorded.
   6. When the group has finished the scoring, ask further questions to check that scores are clear. Follow up any interesting score or relationships with open and probing questions.

Note – the seasonal calendar is a visual method. Do not ask the group to count the counters when distributing them across the months. Instead, ask them to use the counters to show the “pattern” of how the indicator varies by month.

1. Scoring other indicators
   1. Leave the counters for Rainfall in place – do not remove them.
   2. Select another indicator, and explain the diagram for this indicator. Check that the informants understand the meaning.
   3. Take another bag of counters and ask the group to distribute the counters against the months for the indicator.
   4. Again, give them enough time to distribute the counters and don’t interfere. Listen to their discussion as they do the scoring, and record any key points.
   5. When the scoring has finished, ask questions to check the scores, and explain the reasoning behind the scores.

Repeat this process for each of the following indicators:

| Indicator | Explanation | Examples of key questions |
| --- | --- | --- |
| 1. Rainfall | Pattern of rainfall by month | No key questions. |
| 2. Availability of cow milk for children | This indicator is self explanatory, and aims to show the variation in milk supply during the year. | What happens for poor households with no cows, or very few cows? |
| 3. Availability of goat milk for children | This indicator is self explanatory, and aims to show the variation in milk supply during the year. | What happens for poor households with no goat, or very few goats? |
| 4. Availability of own sorghum (Karamoja) or maize (Pokot) | This relates to own production of cereals, and the availability of these cereals for home consumption. | Is all of the sorghum/maize grown in the gardens eaten at home? Is any of the sorghum/maize sold? Is any of the sorghum used for brewing? |
| 5. Women’s work in own gardens | To include land preparation, planting, weeding, harvest etc. | For months when workload is very high, how many hours/day do women spend in the gardens?  For women who are breast feeding, how do they manage the child at this time? |
| 6. Women’s other work | This covers activities related to income generation, other farming activities and so on. | Same or similar to above. |
| 7. Occurrence of child acute malnutrition | This indicator should show when most cases of child acute malnutrition are seen | Why does child AM have this pattern across the months? |
| 8. Occurrence of child diseases – malaria | This indicator should show when most cases of child malaria are seen. | Why does child malaria have this pattern across the months? |
| 9. Occurrence of child diarrhoea | This indicator should show when most cases of child diarrhea are seen. | Why does child diarrhea have this pattern across the months? |
| 10. Births | This indicator should show when most births occur during the year. Are births distributed evenly across the months, or, are there certain months with more births? | Why do most births happen during these months?  Look carefully at the pattern of births against the availability of foods, and women’s labor demands. Does the timings of births match good availability of food, especially milk? Does it match low labor demands? If not, discuss and probe these issues. *What is the ideal time to give birth relative to the availability of milk and labor demands?* |

Note – the examples of key questions are mostly open questions. They use words such as “Why?”, “How?” to open up discussion.

*Supplementary proportional piling*

Related to indicators 2, 3 and 4 in the seasonal calendar, a quick proportional piling can be used to gather further information.

Indicators 2 and 3, about animal milk: *“In the last year, what was the pattern of households with enough access to milk vs. households with insufficient access to milk?”*

1. Repeating the method

In the malnutrition study, the seasonal calendar will be repeated with different groups of women in different locations.

- 1. When repeating the method, it is important that:
- the same diagrams are used each time to show the months and the indicators
- the same indicators are used
- the same numbers of counters are used.
  1. When asking questions to check the information in the seasonal calendar, these questions can vary from place to place.

1. Recording the information

Three types of information need to be recorded:

- 1. When the group is scoring each item, listen to their discussion and record any key or interesting points e.g. their reasons for placing the counters in a particular way.
  2. Record all of the scores against the indicators and months.
  3. With the follow up questions, record the questions asked and the responses.

# Figure S1. Example of a monthly calendar being prepared.


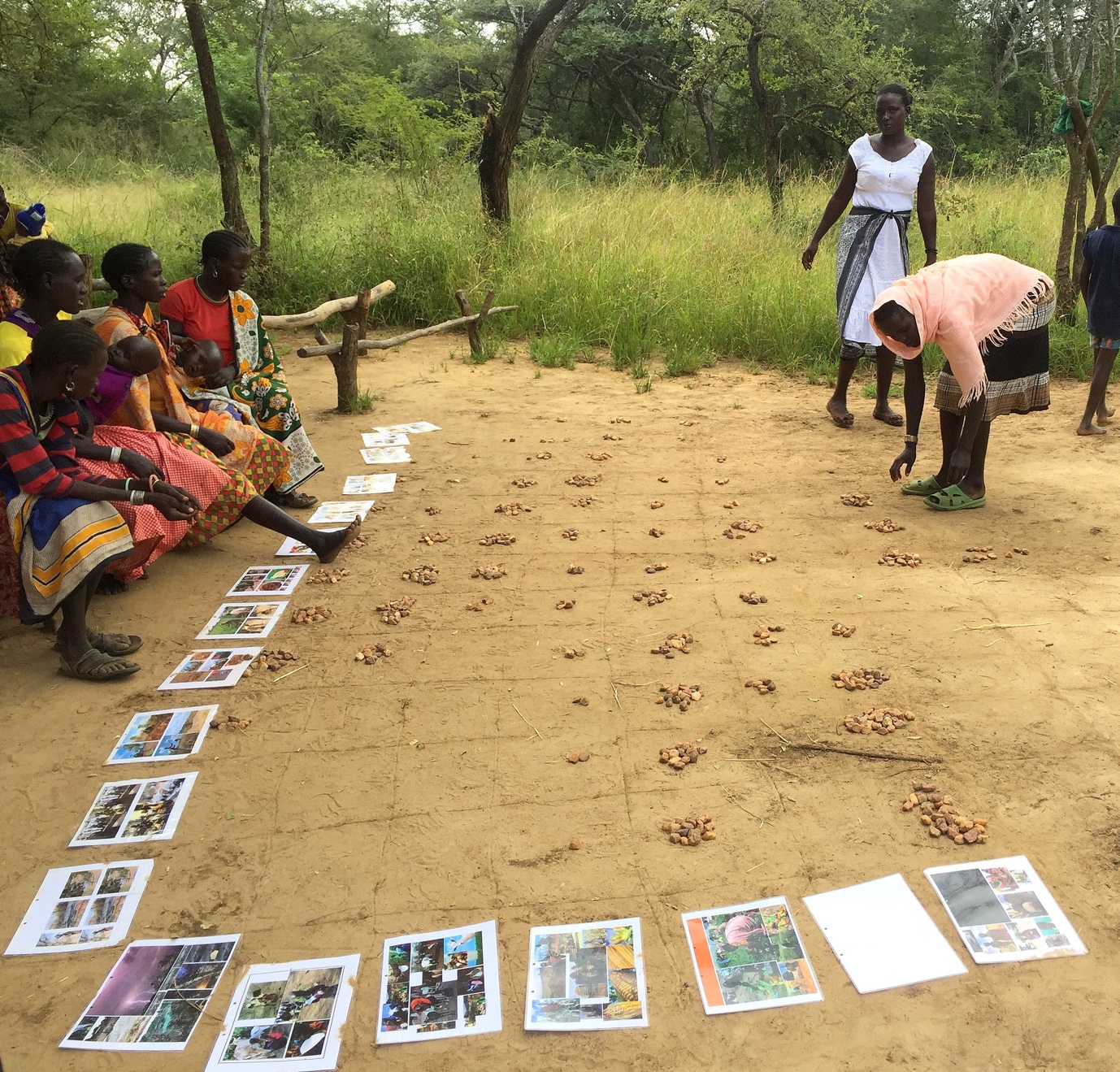


# Table S1. Ngakarimojong language for children and adults by age and gender

| Description | Moroto District | Kaabong District | Kotido District |
| --- | --- | --- | --- |
| Girl  (0–59 months) | *Akaeon* | *Ipethe, ngakipi, itokeng* | *Nyipethe, nyinaas* (nurse) |
| Girl  (5 years and above) | *Apese* | *Ipethe* | *Nyapethe* |
| Boy  (0–59 months) | 0–5 months:  *Ikoku, nika ekisina, iperomoding*  6–9 months:  *Ikoku ni ageiki ekimuje*  10 months–5 years:  *Ikoku ngini imuji bon* | 0–5 months:  *Muzee, idia, ithapat, apakeng, ikoku ngini eroko enaki, nyingwee*  6–9 months:  *Ikoku ngini imuji tanaki, aburianiki, aliya*  10 months–5 years: *Ikoku ni imuji bon, itagae, arotoki* | 0–59 months: *Nyethapat, idokto* (doctor) |
| Boy  (5 years and above) | *Isapat* | *Idia* | *Nyethapat* |

# Table S2. Ngakarimojong language for child size and growth

| Description | Moroto District | Kaabong District | Kotido District |
| --- | --- | --- | --- |
| Child is growing well | *Ikoku ni elosi akwan ejok* | *Ikoku ngini etumit, ikoku ngini engale jik* | *Ikoku ngini ejok akwan, ngini ulap, ngini ulak* |
| Retarded body growth but feeding well | *Ikoku nipe nyepoloe ejok kimuji ejok (tobong akwan kahu)* | *Ikoku ngini korod* | *Ikoku ngini egogong ngakoyo* |
| Feeding well but slim (due to parent’s genetics) | *Ediim akwan* | *Ekotor* | *Ikoku ngini diim, ngini kurab akwan* |
| Strong (due to parent’s genetics) | *Ebutur* | *Etumit* | *Ikoku ngini angikup, Ikoku ngini anginerin* |
| Child with stunted growth | *Ikoku nithirij akwan* (before 5 years) *Ikabuut/iteenge* (after 5 years) | *Nyakibobwang, nyabuth angitunga, nyikaliwoliwot, ikoku niwuriyen, ikoku nibabwi, tukulung, ikoku nitekeny, ikoku niketer, eonit kiu* | *Ikoku ngini babui, ngini gonge, ngini kurab angakoyo, ngini itiitii, ngini euruana* |
| Underweight child |  | *Ikoku ni edoun eroko ngirwa* | *Kutete, ikoku ni eridarit atokeng, ikoku ngini ilongulongunitae* |
| Child born with physical deformities |  | *Emunaara* | *Engwalit* |

# Table S3. Ngakarimojong language for children with different health conditions and different causes of malnutrition

| Description | Moroto District | Kaabong District | Kotido District |
| --- | --- | --- | --- |
| A healthy child from birth to 5 years | *Ikoku ni epolouni ka ngatameta keng ejoak, ikoku ni emam eter ngolo etapit, ikoku ni emam ngatiokisio na etapito* | *Ikoku ni eyerit,*  *ikoku ni etumit* | *Ikoku ngini enaikinit, ikoku ngini etumit, ikoku ngini emam nyichanchani akwan* |
| Unhealthy child—born healthy but becomes unhealthy later | *Ikoku ngini edeakana, ikoku ngini kichiongakin akwan alongaren, ikoku ni etukuruwo edeke ekimuje* | *Ikoku ngini ekara, ikoku ngini kadekan, ekara, ajaka edeke, aditawu ikoku* | *Ikoku ngini adekan, ikoku ngini kekaran, ikoku ngini epeaka ngaakot* |
| Malnourished due to poor diet | *Ikoku nika erogo/ikoku ni angopiki akimuj naajokon imuji* |  | *Ikoku ngini imuji apei kimuj* |
| Malnourished due to food insufficiency |  | *Akoro, ikoku ngini ekadit akimuj* | *Ikoku ngini imuji apei kimuj* |
| Malnourished due to disease | *Ikoku ni amunaaya loitakel*  Stage 1 of being unhealthy: *Ikoku ni adekakin*  Stage 2 of being unhealthy: *Ikoku ni echwangakin akwan*  Stage 3 of being unhealthy: *Ikoku ni amunaiar* | *Ikoku ngini ethikar edeke*  Stage 1 of being unhealthy:  *Ekara*  Stage 2 of being unhealthy:  *Ikadekan*  Stage 3 of being unhealthy:  *Abothia* | *Ikoku ngini ka edeke*  Stage 1 of being unhealthy: *Ikoku ngini adekan*  Stage 2 of being unhealthy: *Ikoku ngini ebelok edeke*  Stage 3 of being unhealthy: *Ikoku ngini ekarun* (given up on the child) |
| Malnourished due to non-spaced pregnancies | *Ikoku nika eeter* | *Tutukan/itungi* | *Ikoku ngini a nyeneto* |
| Malnourished due to poor quality of breast milk | *Ikoku ni epeaka ngakile atotokeng* | *Epeaka ngakile aitoke* | *Ikoku ngini epeaka atokeng ngakile angikithin* |
| Malnourished due to inadequate breast milk | *Ikoku ni ekadito ngakile angisikin* | *Ekadito ngakile atotokeng* | *Ngini euchuriana atokeng* |
| Malnourished due to poor relationship between father and mother |  | *Ni angichan, ni achaka apakeng* |  |

# Table S4. Ngakarimojong language for mothers at different stages of pregnancy, at birth, at different stages of lactation

| Description | Moroto District | Kaabong District | Kotido District |
| --- | --- | --- | --- |
| 2 weeks to 3 months | *Aberu na eweiyarit* | *Irumiyarit, epotiyorit, ekama akook* | *Aberu ngina irumiarit* |
| 4–6 months | *Aberu na awua ngaakot* | *Atakanu akook, apotiyo* | *Aberu ngina epoti* |
| 7–9 months | *Aguothu/aberu na adol akidoun* | *Agothu akook* | *Aberu ngina adou* |
| Has just given birth | *Aberu na alek* | *Alek; engarakin* |  |
| Still in the house—up to 1 to 2 weeks | *Aberu na erongo nyipuduno kinga* | *Eyei kai, imanyurit* |  |
| Umbilical cord of the baby not yet cut | *Aberu na erongo nyedengoro* |  |  |
| Out of the house—after 1 to 2 weeks | *Aberu ngina epudun kinga/aberu ngina elotun* | *Apudu alokai* |  |
| 0–9 months (sometimes up to 2 years) | *Aberu ngina itanaki* | *Itanaki* | *Aberu ngina manangit, ngina ketanakan* |
| 9 months–2 years (may not be breastfeeding anymore) | *Aberu ngina apeto* | *Apeto* | *Aberu ngina apeto* |
| Breastfeeding beyond 2 years |  |  | *Aberu ngina kori, ngina ewoyakinit* |

# Table S5. Ngakarimojong language for pregnant mothers with different health conditions and different causes of malnutrition

| Description | Moroto District | Kaabong District | Kotido District |
| --- | --- | --- | --- |
| Healthy pregnant women due to good diet | *Aberu na epoti tangalee, aberu na epoti kimuji ejok* | *Aberu ngina epoti totumite, na eyarit noi, engale akekwaan, kejoiki apot* | *Aberu ngini ebene akekwan* |
| Healthy due to lack of disease | *Aberu ngina epoti tamam edeke* |  |  |
| Malnourished/weak due to lack of appetite | *Aberu na itengerioritae ekimuje/aberu na itengeriorit apot kori ngaakot aikoku ekimuje* | *Imakuwat, ekara, edwarikinit* | *Aberu ngina edwarikinit apoti, ngina inuikinit* |
| Malnourished/weak due to lack of appetite because of disease | *Aberu na itengeriorit edeke ekimuje* |  |  |
| Malnourished/weak due to food shortage | *Aberu na engopikinit akimuj* | *Ekadit akimuj* |  |
| Malnourished/weak due to disease | *Aberu na epoti todeaka* | *Edeak* |  |

# Table S6. Women’s activities and times in own gardens by month

| Month | Activity and times |
| --- | --- |
| January | Land preparation—clearing bushes for new gardens or cleaning the existing gardens   - 6 am to 12 pm then 3 pm to 6 pm - 7 am to 4 pm or up to 6 pm - 7 am to 12 pm - 8 am to 12 pm or up to 1 pm |
| February | Gathering and burning waste from the cleared bushes and previous harvest   - 7 am to 10 am - 6 am to 1 pm |
| March | Digging with hand hoes   - 7 am to 2 pm - 7 am to 3 pm or up to 5 pm - 5 am to 3 pm |
| April | Ox-ploughing; planting sorghum and some beans, maize and millet   - 7 pm to 1 am then 4 am to 12 pm or up to 3 pm - 3 am to 11 am or up to 1 pm |
| May | Weeding   - 7 am to 2/3/4 pm or up to 6 pm |
| June | Final weeding; harvesting pumpkins, vegetables and beans; scaring birds and cows/goats from gardens   - 7 am to 1 pm; 6 am to 6 pm |
| July | Harvesting millet, white sorghum and beans (not much); harvesting melons, pumpkins and wild/local vegetables; scaring birds, monkeys and baboons from gardens |
| August | Harvesting white sorghum; harvesting red sorghum fallen by frogs, armyworm/butterflies and rain; scaring birds   - 7 am to 11 am |
| September | Some red sorghum ready and harvested; construction of granaries |
| October | Intensive harvesting of red sorghum, heaping on racks on farms, threshing and winnowing, and storing in granaries   - 7 am to 2 pm or up to 3 pm or up to 6 pm |
| November | Threshing and winnowing sorghum; construction of houses   - 7 am to 4 pm |
| December | Construction of houses |

# Table S7. Illustrative women’s work activities and time spent

| Activity | Agro-pastoralist areas |
| --- | --- |
| Aloe vera harvesting processing | 8am to 6pm |
| Working in mines | 4am to 5pm |
| Casual labour in towns (cooking or selling food in restaurants, cooking in homes, fetching water, washing clothes) | 6am to 6pm |
| Casual labour in other people’s gardens—clearing gardens, digging, planting, weeding, harvesting/heaping/storage of grain | 7am to 1pm |
| Sale of firewood and charcoal | Charcoal burning and selling—takes up to three weeks to get a batch for sale  Firewood collection and sale—collection (4 am to 11 am or up to 1/2/3/4 pm depending on distance; selling—5 am to 12 pm or sometimes longer if demand is low |

**Table S8. Ngakarimojong language for months**

| English name | Ngikarimojong |
| --- | --- |
| January | *Lokwang* |
| February | *Lodunge* |
| March | *Lomaruk* |
| April | *Titima* |
| May | *Yeliyel* |
| June | *Lomodokogech* |
| July | *Losuban* |
| August | *Lotiak* |
| September | *Lolobai* |
| October | *Lopoo* |
| November | *Lorara* |
| December | *Lomuk* |
